# Left Ventricular Apex: A “Minimally Invasive Motorway” for Safe Cardiovascular Procedures

**DOI:** 10.3390/jcm10173857

**Published:** 2021-08-27

**Authors:** Andrea Agostinelli, Alan Gallingani, Francesco Maestri, Silvia Grossi, Florida Gripshi, Luca De Donno, Francesco Nicolini

**Affiliations:** 1 Cardiac Surgery Division, Parma University Hospital, Via Gramsci 14, 43126 Parma, Italy; agallingani@ao.pr.it (A.G.); fmaestri@ao.pr.it (F.M.); florida.gripshi@gmail.com (F.G.); luca.dedonno@unipr.it (L.D.D.); francesco.nicolini@unipr.it (F.N.); 2 Cardiac Surgery Intensive Care Unit, Parma University Hospital, Via Gramsci 14, 43126 Parma, Italy; sigrossi@ao.pr.it

**Keywords:** transapical approach, transapical TAVR, transapical TEVAR, transapical mitral valve repair, transcatheter mitral valve replacement, transapical paravalvular leak repair

## Abstract

Since the advent of TAVR (transcatheter aortic valve replacement), the transapical surgical approach has been affirmed as a safe and effective alternative access for patients with unsuitable peripheral arteries. With the improvement of devices for transfemoral approach and the development of other alternative accesses, the number of transapical procedures has decreased significantly worldwide. The left ventricular apex, however, has proved to be a safe and valid alternative access for various other structural heart procedures such as mitral valve repair, mitral valve-in-valve or valve-in-ring replacement, transcatheter mitral valve replacement (TMVR), transcatheter mitral paravalvular leak repair, and thoracic aorta endovascular repair (TEVAR). We review the literature and our experience of various hybrid transcatheter structural heart procedures using the transapical surgical approach and discuss pros and cons.

## 1. Introduction

Transcatheter Aortic Valve Replacement (TAVR) was introduced for the treatment of aortic valve stenosis in 2002 by Cribier [1], and the transapical approach was first described by Lichtenstein et al. in 2006 [2]. The transapical approach then became the main alternative route for TAVR in patients deemed at high surgical risk due to significant comorbidities and/or technical aspect such as porcelain aorta, hostile thorax or previous surgical coronary artery revascularisation with mammary artery grafts running underneath the sternum, and with poor peripheral accesses.

In recent years, unfavourable outcomes have been reported, and reduction of sheath calibres for the transfemoral approach, together with the development of other alternative endovascular procedures, have led to a fall in the number of transapical TAVR procedures performed worldwide.

Despite this, some researchers have developed consistent experience with the left ventricular apex manipulation and reported satisfactory results in large series of transapical TAVR. The left ventricular apex proved to allow safe access and had great advantages: short distance and good accessibility with good coaxiality with multiple heart structures, and thus good stability of the devices during implantation. For all these reasons, the trans-apical approach can thus also be a useful tool for multiple transcatheter structural heart procedures other than TAVR: transcatheter mitral valve-in-valve and valve-in-ring replacement, mitral valvuloplasty, transcatheter mitral valve replacement (TMVR), paravalvular leak occlusion, and thoracic endovascular aortic repair (TEVAR). Left ventricular outflow tract pseudo-aneurysm occlusion is also described.

## 2. Transapical TAVR

**Figure 1:**
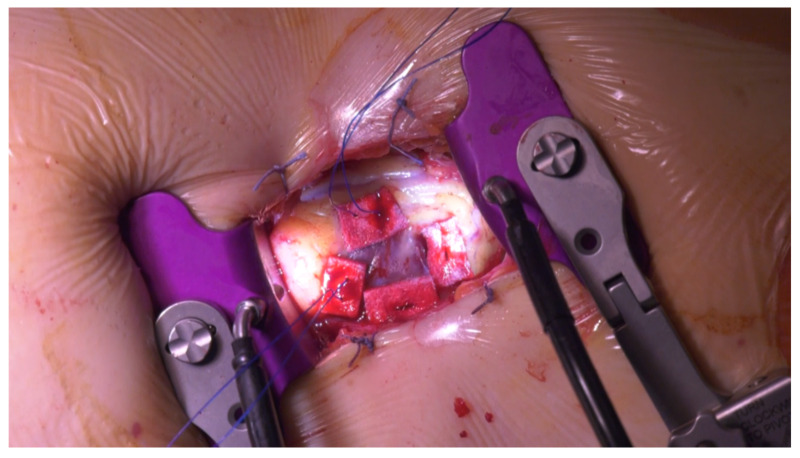
Two haemostatic orthogonal U-shaped 2/0 polypropylene stitches on the left ventricular apex.

Transapical TAVR was introduced into clinical practice to treat patients deemed at high surgical risk for standard surgical aortic valve replacement and also at high risk of vascular complications for the transfemoral approach due to peripheral arteries occlusive disease, calcifications or excessive tortuosity. Transapical TAVR is a hybrid procedure in which arterial access for a transcatheter aortic valve implantation is obtained by surgical isolation of the left ventricular apex through a standard left anterolateral 5–7-cm-long thoracotomy in the fifth or sixth intercostal space. After opening the chest, the correct location of the apex is identified with the “finger test”: under transoesophageal echocardiography (TEE) guidance, the left ventricular apex is pushed with the finger to determine at the four chambers view the ideal place to insert the sheath in order to obtain the best possible coaxiality. We place two orthogonal full-thickness U-shaped 2–0 polypropylene stitches on the muscular portion of the myocardium to assure correct haemostasis after sheath removal (Figure 1). At this point a standard transcatheter procedure under ventricular pacing is performed.

Since the early days of TAVR, the transfemoral approach has been considered the treatment of choice, and patients undergoing transapical TAVR have concomitant severe peripheral artery occlusive disease and tend to be sicker and with a higher surgical risk profile due to significant comorbidities [2]. Several groups have reported large series of patients with acceptable results compared to those predicted by surgical risk scores. In 2011 D’Onofrio et al. reported the results of 504 “all comers” from the Italian Registry of Trans-Apical Aortic Valve Implantation (I-TA). The overall 30-day mortality rate was 8.7%, cardiovascular mortality 6.7% and stroke incidence was 3% [3]. In the same study, 1-, 2- and 3-year survival rates were 81.7%, 76.1% and 67.6% [4]. Thourani in 2015 presented his experience of 4085 patients with an early mortality rate of 6.7% in a population of high-risk patients [5].

The “transfemoral first” approach has become more widespread over the years, and several randomised trials reported a consistent gap in terms of survival and adverse outcomes in favour of transfemoral over transapical TAVR [6,7,8]. These are large multi-centre studies involving numerous different sites and surgical equipes. There are how-ever no randomised trials comparing the transfemoral and transapical approaches directly. Blackstone et al. in a PRTNER-1 substudy identified and compared 501 matched pairs of patients submitted to transapical or transfemoral TAVR with a balloon-expandable bioprosthesis on the basis of 111 preprocedural variables. They concluded that periprocedural adverse events and prolonged recovery were higher in the transapical group, while stroke risk was the same and aortic regurgitation was lower. They thus confirmed the recommendation for a ‘transfemoral first’ approach when feasible.

Considering that patients submitted to transapical TAVR are usually affected by significative peripheral artery occlusive disease, they tend to also be sicker due to other significant comorbidities [9]. For this reason, worse outcomes compared to those of transfemoral TAVR could be not only related to the kind of approach itself.

Over time, however, many centres gained valuable experience in managing the left ventricular apex and thanks to improvements in materials, were able to report satisfactory results for the transapical approach in patients unsuitable for the transfemoral procedure [10]. Some large propensity-score matching series with the transfemoral approach in fact showed no statistical difference in survival between the two accesses. Ferrari et al. compared 90 consecutive patients undergoing transapical TAVR with 90 consecutive patients undergoing transfemoral TAVR between 2009 and 2014 at their centre, and found similar mortality rates and adverse neurological events even though patients in the transapical group had a much higher preoperative surgical risk. Mild to severe postoperative aortic regurgitation prevalence was higher in the transfemoral group [11]. The same conclusions were drawn in 2015 by Schymik et al. reporting outcomes of propensity matched groups of 354 patients undergoing both transapical and transfemoral approaches out of a real-world population of 1000 patients with aortic stenosis. Schymik et al. found there was no difference in early and long-term adverse event rates between the two groups, and concluded that in experienced centres with a multidisciplinary heart team, either access route can be used with comparable results [12]. According to a meta-analysis of direct and adjusted indirect comparison of early and mid-term results of patients undergoing the two approaches conducted by Ando et al. in 2017, early mortality did not differ in the two groups while mid-term survival was higher in the transfemoral group after combined direct and indirect meta-analysis [13]. In 2019 Reents et al., after a multivariate logistic regression analysis of early and mid-term outcomes of transapical and transfemoral approach performed at their centre between 2009 and 2016, concluded that TAVR access route was not associated with a higher mortality rate. Only surgical risk profile (Logistic EUROSCORE) and institutional experience were found to be risk factors for higher mortality [14]. In Table 1 we summarise the results of the studies comparing TA and TF TAVR.

In the early days, larger sheaths were used, and access site complications were re-ported. In 2011 Bleiziffer et al. reported a rate of 7% of severe apical bleeding and 1% (2 patients) of apical pseudo-aneurysm, in one case requiring surgical revision, on a series of 143 patients undergoing transapical TEVAR [16]. This led to the belief that the transapical approach was unfit for low-ejection fraction patients. Recently, however, favourable outcomes have been reported even in this particular subsetting [17,18,19]. Left anterior descending (LAD) coronary artery injury can also occur suturing the left ventricular apex; precise identification of the LAD during the procedure by means of direct vision is mandatory to prevent this complication.

Our TAVR programme with a “transfemoral first approach” was started in 2009, and to date the multidisciplinary team has remained the same. Our preliminary results were collected in the Italian Registry of Transapical Aortic Valve Implantation and published by D’Onofrio and coll [3,4]. Up to May 2021, we performed 189 transapical TAVR in high-surgical-risk patients with poor peripheral access due to unfit diameters, calcifications, excessive tortuosity of iliac–femoral axes, or severe pathology of the thoracoabdominal aorta. Two dedicated surgeons, and one dedicated cardiologist, performed all the procedures, together with the only nurse trained for valve crimping and preparing. The early mortality rate was 4.76%, the stroke rate was 1.08%, and there was no moderate to severe aortic paravalvular leaks.

According by the AHA/ACC focused update of the 2014 guidelines TAVR should be reserved for inoperable/high-risk patients (Class I recommendation) and intermediate-risk patients (Class IIa reccomendation) as defined by the STS score. The guidelines also state that a multi-disciplinary heart team approach is mandatory to define the risk profile of each patient and subsequently select the most appropriate procedure [20].

## 3. Transapical Transcatheter Mitral Procedures

Transcatheter mitral procedures can be performed with a venous transeptal endovascular antegrade approach, or a with a direct retrograde approach through the left ventricular apex isolated with a standard anterolateral minithoracotomy. As described for TAVR, in the transapical approach, the distance from the left ventricular apex to the mitral annulus is very short. Together with coaxiality of the catheter with mitral anulus, this guarantees easy access and high stability during implantation.

### 3.1. Mitral Transcatheter Valve-in-Valve and Valve-in-Ring Replacement

After initial experience with transapical TAVR, surgeons started to report transcatheter transapical mitral valve replacement for degenerated mitral bioprosthesis and failed mitral valve repairs in which an anuloplasty prosthetic ring was used to reinforce the repair during the first intervention [21,22,23]. The metallic stent of the biological prosthesis and anuloplasty ring proved from the beginning to be a solid anchorage site for the transcatheter valves. The weakness of the procedure could be left ventricular outflow tract (LVOT) obstruction by the transcatheter valve itself, and most frequently by the native mitral valve anterior leaflet dislocation towards it. Of course, this complication is less likely in valve-in-valve (ViV) procedures, and in any case can be avoided by making an accurate evaluation during the preoperative computerised tomography (CT) scan. As far back as 2015, Conradi et al. evaluated the results of 75 transcatheter ViV procedures in four different anatomic positions [24]. Mitral ViV procedures were performed in 17 high-risk redo patients (22%) and transapical access was used in 40 (53.3%). The elective 30-day mortality rate in Mitral ViV was 12.5%. The mean gradient was 4.7 mm Hg and no cases of more than mild paravalvular leak were registered. Conradi et al. concluded that transcatheter ViV procedures can be safely performed on high surgical risk patients. Data from the Society of the Thoracic Surgeons (STS) and the American College of Cardiology (ACC) Transcatheter Valve Therapy (TVT) registry on mitral ViV, valve-in-ring and valve in mitral annular calcification (MAC) showed the feasibility and safety of the procedure in high-risk patients, with a nearly 88% success rate, and a rate of only 2.3% of LVOT obstruction [25,26]. Transapical access was chosen in 44.8% of the patients. The mortality rate was lower than that predicted by the STS mortality score. The success rate was lower and mortality rates and LVOT obstruction rates were higher in the valve-in-MAC group.

Some authors report similar early and one-year clinical results among patients undergoing trans-septal, transapical mitral transcatheter ViV procedures and open surgical re-mitral valve replacement [27], while others find that the transeptal transcatheter approach is associated with lower overall 1-year mortality rates [28].

### 3.2. Transcatheter Native Mitral Valve Replacement

There have been attempts to treat mitral stenosis according to the same principles and with the same devices as TAVR [29], and many devices have been developed for transcatheter mitral valve replacement in mitral regurgitation and are currently under clinical investigation. Most of these devices use transapical delivery systems. They have been implanted in patients deemed at high or prohibitive surgical risk for open surgical replacement of the valve.

Systematic reviews of results reported in published studies and international conference presentations conclude that TMVR is a feasible alternative to open mitral replacement in high-risk patients [30,31]. These reviews report a high success rate of implantation and optimal haemodynamics with low transvalvular gradients and no significant residual regurgitation, albeit with relatively high 30-day mortality rates. Chen et al. also observed that transapical access (62%) was associated with higher technical implant success due to better anchoring systems [31]. Of course, the number of patients is still very low, and many studies report relatively inexperienced centres using different devices, which may explain the conflicting results. Bapat et al. prospectively collected and reported outcomes of 50 consecutive patients considered inoperable and undergoing TMVR with a self-expanding valve (Intrepid TMVR System, Medtronic Inc, Redwood City, CA, USA) at 14 different hospitals [32]. The success rate of implantation was 98% and the rate of early mortality was 14%.

Promising results were reported by Muller et al. in the global feasibility trial of the Tendyne Mitral Valve System (Abbott Vascular, Roseville, MN, USA) [33]. The valve system consists of a self-expandable porcine valve and includes a tether anchoring it to an epicardial pad and holding it into position. In the global feasibility trial on 30 patients across eight centres worldwide, the successful implantation rate was 93.3%, the 30-day mortality rate was 3.3%, and the mean postoperative transvalvular gradient was 3.4 mm hg with no moderate or severe postoperative mitral regurgitation. There were no acute strokes or myocardial infarctions. In 2019 the early and 1-year outcomes of the first 100 patients of the feasibility study were published by Sorajja et al. [34]. They reported a success rate of 96% with no acute death or conversion to surgery and 6% and 2% 30-day rates of mortality and strokes, respectively. The 1-year survival rate, free of all-cause mortality, was 72.4%. The Tendyne Mitral Valve System is the first commercially available valve in Europe.

Further studies with larger series of patients and other devices are necessary to better evaluate and validate the procedure

### 3.3. Transapical Mitral Valve Repair

Several devices for transcatheter mitral valve repair are nowadays commercially available. Most of them use the transvenous transeptal approach.

Nechord DS100 (Neochord, Eden Prairie, MN, USA) is a device that allows artificial chord implantation on prolapsing mitral valve leaflets using the transapical approach on a beating heart without cardiopulmonary bypass. The surgical technique is described step by step by Colli et al. [35]. The chords are fixed to the epicardial surface of the ventricle. The delivery system is inserted into the left ventricular apex isolated by way of a standard left anterolateral minithoracotomy. Transoesophageal echocardiography guidance is of paramount importance in order to identify the exact spot in the ventricle to insert the chords, and to determine the level of tension to apply to the chords themselves in order to obtain the optimal leaflets coaptation.

Ahmed et al. systematically reviewed the published literature on transapical beating-heart mitral valve repair with Neochord [36], and identified six studies covering a total of 249 patients. The operative success rate was 96.8% and the 30-day mortality rate was 1.9%. Significant bleeding from the left ventricular apex was recorded in 3.2% of the patients. At follow-up, the effect was maintained in 100% of the cases in only one study. Ahmed et al. noted that the best 1-year outcomes were associated with stricter patient selection. Moreover, patients with only posterior leaflet prolapse also appear to have better outcomes than patients with other associated lesions. Ahmed et al. concluded that, while awaiting results of larger studies, transapical off-pump repair with NeoChord implantation is a promising procedure that can be performed in carefully selected cases and in experienced centres. Several preclinical programs are under development to move from transapical towards a fully transcatheter procedure that could represent a valid alternative to transcatheter edge to edge mitral repair. Further studies directly comparing the techniques are mandatory to draw any conclusion.

### 3.4. Transapical Mitral Paravalvular Leak Repair

**Figure 2:**
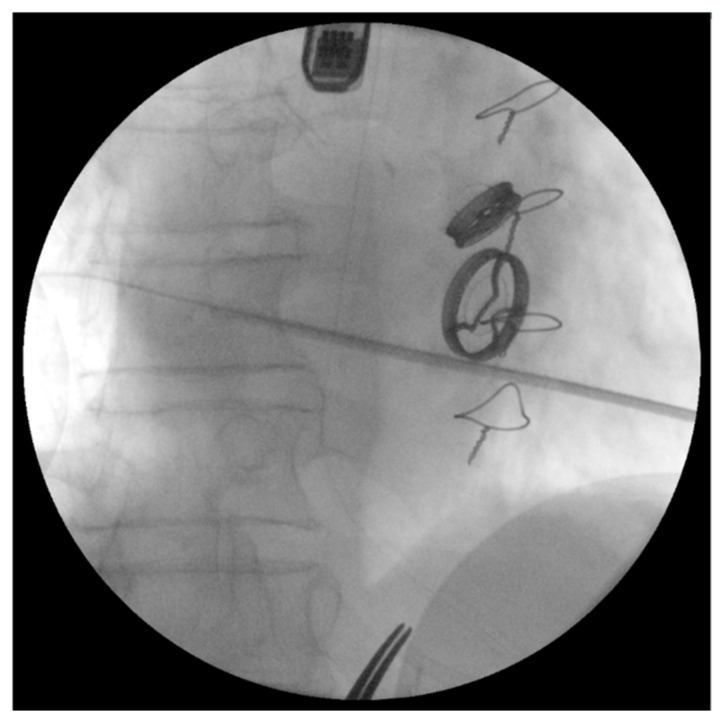
Easy engagement of mitral postero-medial paravalvular leak.

Thranscatheter endovascular paravalvular leak (PVL) repair has become a reasonable alternative to open surgical reintervention for high risk patients. The procedure is performed with either arterial or venous femoral access, but in any case, it is technically demanding. The transapical approach offers a very easy alternative, although it involves surgical minithoracotomy and general anaesthesia. The transapical route guarantees direct access to the mitral annulus and an easy engagement of PVL, even those in the postero-medial position for which transvenous and transarterial approaches are particularly challenging (Figure 2).

Some surgeons find that transapical access is associated with lower procedural and fluoroscopy time than other approaches [37].

In 2014 Taramasso et al. report results of 139 patients undergoing open surgical (122 patients) and hybrid transapical transcatheter (17 patients) mitral paravalvular leak occlusion [38]. The overall procedural success rate was 98% (98% in the surgical group and 94% in the transapical group). The hospital mortality rate was 9.3% in the surgical group, while no acute deaths were registered in the transapical group. Univariate analysis shows that surgical procedure was a risk factor for in hospital death. The devices used to address the PVL from the left ventricular apex were Amplatzer Vascular Plugs III and II (Abbott Vascular, Roseville, MN, USA).

Zorinas et al. review their single-centre experience of 19 patients undergoing transapical mitral PVL closure with the novel specifically designed device Occlutech PLD Occluder (Occlutech, Jena, Germany) [39]. They report no early death, strokes or myocardial infarctions and a 95% reduction in mild or lesser paravalvular regurgitation.

In 2020 Onorato et al. report midterm results of 136 patients undergoing aortic or mitral PVL transcatheter closure in 21 sites in 9 countries with the same device (Occlutech PLD Occluder) [40]. Access routes were transapical, transarterial and transvenous. Onorato et al. report a fall in the proportion of patients in New York Heart Association (NYHA) class III/IV from 77.3% to 16.9% at follow-up.

## 4. Transapical TEVAR

TEVAR is nowadays the treatment of choice for many acute aortic syndromes and elective aortic pathologies. Deployment of stent grafts is usually retrograde, through femoral and iliac arteries. Delivery sheath and catheter calibres range from 14 to 24 French and require large minimal inner arterial diameters. Thoracic pathologies or acute aortic syndromes often affect elderly patients with peripheral arterial occlusive disease, which precludes standard retrograde endovascular treatment. Alternative approaches, like common iliac arteries or infrarenal abdominal aorta, are often required. After reporting the feasibility of the technique in a pig model [41], MacDonald et al. performed the first antegrade TEVAR through the left ventricular apex of a beating heart in 2009 [42]. Other researchers report feasibility and advantages of this technique [43].

**Figure 3:**
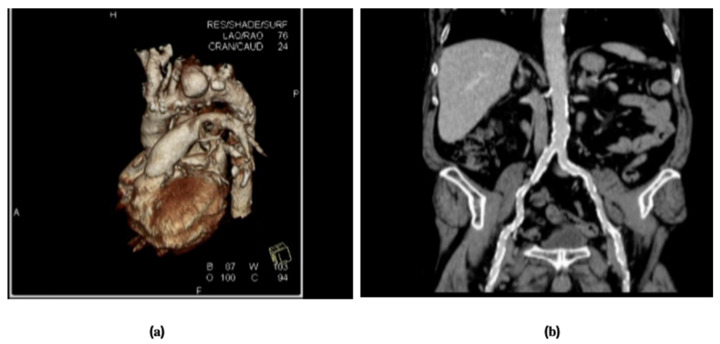
Pre-operative CT-scan: (**a**) 3D reconstruction of distal arch pseudo aneurysms due to penetrating aortic ulcers; (**b**) coronal view showing severe calcific occlusive disease of infra-renal aorta and peripheral arteries.

Our transapical TAVR program started in 2010, and we described our first case of a successful transapical TEVAR of a patient admitted for an acute aortic syndrome in 2012 [44]. He was an 83-year-old male with symptomatic acute penetrating aortic ulcer in the distal portion of the aortic arch with severe concomitant calcific occlusive disease of the aorto-iliac femoral axes, which precluded a retrograde standard TEVAR (Figure 3a,b).

Antegrade delivery of the thoracic stent graft through a left anterolateral minithoracotomy was performed. A Dry-Seal 22 French sheath (W.L. Gore & Assoc, Flagstaff, AZ, USA) was inserted into the left ventricular apex. The aortic valve was first inspected through trans-thoracic and trans-oesophageal echocardiography and presented no stenosis or regurgitation. Exclusion of the lesion was achieved, and the patient discharged on post-operative day 15.

In 2018 we then reported a series of five patients affected by acute aortic syndromes undergoing transapical TEVAR: two patients presented with a post-traumatic aortic injury with signs of impending rupture, two patients with contained aneurysmatic aortic rupture and one patient with symptomatic PAU with large pseudo-aneurysm [45]. All patients had poor peripheral access and were thus considered unsuitable for the standard retrograde approach. Successful treatment of the lesion was achieved in all cases and no damage to the aortic valve was reported.

We also performed an elective transapical TEVAR as completion of a previous complete aortic arch replacement using the Elephant Trunk (ET) technique on a patient with extended aneurysmatic disease of the aortic arch and descending aorta, peripheral arteries occlusive disease and severe aortic tortuosity [46].

Other researchers have described percutaneous transapical access to create a rail wire support for a very complex retrograde TEVAR [47]. They inserted a wire in the left ventricular apex, snared into the descending aorta and then externalised from the femoral artery to support the procedure.

The transapical antegrade approach proved to be a feasible option for experienced multidisciplinary teams for patients with complex thoracic aorta pathologies deemed unsuitable for standard retrograde TEVAR due to concomitant occlusive disease of the peripheral vessels. Considering the large size of the sheaths used for stent graft delivery, a preoperative aortic valve assessment with echocardiography is mandatory to exclude calcific aortic disease that could determine an acute impairment of hemodynamic conditions during the procedure.

Transapical closure of left ventricular outflow tract (LVOT) pseudoaneurysm is also described in the literature [48,49].

## 5. Conclusions

Although in recent years the number of transapical procedures has decreased worldwide thanks to the size reduction of sheath calibres in the standard transfemoral approach, the transapical approach continues to be a valid alternative option for procedures normally performed using transfemoral access, such as TAVR and TEVAR, in elderly patients deemed to be at high surgical risk for standard surgical procedures with unsuitable peripheral arteries. The close vicinity and coaxiality of the left ventricular apex with several heart structures such as mitral valve and LVOT makes the transapical approach a safe and easy option that guarantees easy access and great stability and accuracy for several structural procedures.

Possible left anterior descending coronary artery damage, apical bleeding and late left ventricular apical pseudoaneurysm formation are threatening possible complications of the transapical procedures. For this reason, considerable experience in ventricular apex manipulation as well as complications management and a multidisciplinary heart team evaluation are mandatory to properly evaluate patient risk profile, select the appropriate approach and safely perform a transapical procedure.

Further improvements in devices and randomised control trials comparing TF vs. TA and TA vs. standard surgical procedures are necessary to standardise and validate the transapical approach in the subsetting of different structural heart procedures.

We consider the transapical approach a valid and essential tool in the portfolio of a modern heart valve centre.

## Figures and Tables

**Table 1:** Studies comparing results of TA vs. TF TAVR.

	No. of Patients		Hospital Mortality		Neurological Complications		Major Vascular Complications		PPM Implantation		Residual AR (Mild to Severe)	
	TA	TF	TA	TF	TA	TF	TA	TF	TA	TF	TA	TF
Ferrari et al. [11]	90	90	8 (9%)	9 (10%)	2 (2%)	3 (3%)	3 (3%)	10 (11%)	2 (2%)	5 (6%)	5 (6%)	23 (26%)
Schymik et al. [12] ^§^	354	354	21 (5.9%)	30 (8.5%)	7 (2%)	8 (2.3%)	9 (2.5%)	56 (15.8%)	41 (11.6%)	58 (16.4%)	4 (1.1%)	13 (3.7%)
Rents W. et al. [14] *	511	619	34 (6.7%)	30 (4.8%)	12 (2.3%)	10 (1.6%)	48 (9.4%)	57 (9.2%)	94 (18%)	108 (17%)	13 (2.5%)	16 (2.6%)
Biancari et al. [15] ^§^	199	199	8%	4%	2%	1%	1%	7.2%	8.7%	13.3%	//	//
Blackstone et al. [9] ^§^	501	501	9%	3.4%	2.8%	3.2%	3.8%	6%	7.3%	6.5%	//	//

* Non propensity score matched population; ^§^ Articles examined in the meta-analysis published by Ando et al. [13].
